# Psychometric properties of the Dutch WHOQOL-OLD

**DOI:** 10.1186/s12955-016-0508-5

**Published:** 2016-07-15

**Authors:** Robbert J. J. Gobbens, Marcel A. L. M. van Assen

**Affiliations:** Faculty of Health, Sports and Social Work, Inholland University of Applied Sciences, De Boelelaan 1109, 1081 HV, Amsterdam, The Netherlands; Zonnehuisgroep Amstelland, Amstelveen, The Netherlands; Department of Methodology and Statistics, Tilburg School of Social and Behavioral Sciences, Tilburg University, Tilburg, The Netherlands; Department of Sociology, Utrecht University, Utrecht, The Netherlands

## Abstract

**Background:**

To assess the internal consistency reliability and construct validity of the Dutch version of the World Health Organization Quality of Life Instrument-Older Adults Module (WHOQOL-OLD).

**Methods:**

The psychometric properties of the Dutch WHOQOL-OLD were examined in a cross-sectional study using a sample of 1,340 people aged 60 years or older. Participants completed a Web-based questionnaire, the ‘Senioren Barometer’. Reliability was evaluated using Cronbach’s alpha and corrected item-total correlations. Construct validity of the Dutch WHOQOL-OLD was evaluated with confirmatory factor analyses, and correlations within and between scales, using scales WHOQOL-BREF, Short Form Health Survey (SF-12), Tilburg Frailty Indicator (TFI), and the Emotional and Social Loneliness Scale (ESLS).

**Results:**

The reliabilities of the six WHOQOL-OLD facets or subscales were sufficient to good (.66-.91). The convergent validity of the WHOQOL-OLD was good, whereas our findings on the divergent validity of the WHOQOL-OLD were somewhat mixed. Findings corroborating the divergent validity were that the 6-factor model fitted better than the second-order factor model, and WHOQOL-OLD facets sensory abilities, past, present and future activities, death and dying, intimacy correlated more strongly with similar than dissimilar scales. Not fully supporting divergent validity were the extremely high correlations between the factors corresponding to autonomy, past, present and future activities, and social participation.

**Conclusion:**

We offer Dutch healthcare and social workers an instrument with good psychometric properties for measuring quality of life in older people. Further research on interrelations between WHOQOL-OLD facets is recommended.

## Background

The human populations of most countries in the world are ageing rapidly because of declining fertility rates and increasing longevity [1]. Currently, Europe has the highest proportion of older people (24 %) and is projected to reach 34 % in 2050 and 35 % in 2100 [1]. In the Netherlands it is estimated that by 2050 33.2 % and by 2100 36.6 % of the population will be 60 and over [1]. These changing demographics have important implications for Dutch policymakers, as well as professionals providing health and social services. Social, economic, and political conditions result in a great variety in the state of health and living conditions of older people [2]. To support independent living in older people it is necessary for health and social care professionals to carry out interventions focusing on aspects of quality of life, with the aim of delaying hospitalization or institutionalization.

Quality of life has been defined by the World Health Organization Quality of Life (WHOQOL) group as “an individual’s perception of their position in life in the context of the culture and value systems in which they live and in relation to their goals, expectations, standards, and concerns” [3]. The World Health Organization has developed two generic instruments for assessing quality of life, the WHOQOL-100 and its short form the WHOQOL-BREF [4, 5]. However, it became obvious that both instruments were insufficient for the specific requirements of assessing quality of life in old age [2]. Therefore, for older subjects, the WHOQOL Group has decided to develop the World Health Organization Quality of Life Instrument-Older Adults Module (WHOQOL-OLD), following the WHOQOL Group methodology [6], including specific items important to older people referring to autonomy, death and dying, and intimacy. Nowadays, the WHOQOL-OLD has been translated into more than 20 languages, and several studies have reported its reliability and validity [2, 7–13].

Several other instruments have been developed for assessing quality of life in older people, such as the Elderly Quality of Life Index (EQOLI) [14], the WHOQOL-AGE [15], the CASP-19 (Control, Autonomy, Self-realisation, Pleasure) [16] and the Older People’s Quality of Life Questionnaire (OPQOL) [17]. There were two reasons for choosing to examine the psychometric properties of the Dutch version of the WHOQOL-OLD instead of these other instruments. First, the WHOQOL-OLD is the most frequently used instrument, not only in the Netherlands but also in other countries, thus allowing for international and inter-cultural comparisons. Second, the WHOQOL-OLD contains items referring to autonomy, death and dying, and intimacy that are particularly relevant for older people; most of these items are lacking in the other instruments.

Because of the rapidly ageing Dutch population, we believed that a validation of the Dutch WHOQOL-OLD has been long overdue. Therefore, we present the results of a cross-sectional study examining its psychometric properties in a large sample of people aged 60 years or older. The results of an evaluation of the internal consistency reliability and construct validity are presented.

## Methods

### Study population and data collection

The ‘Senioren Barometer’ is a web-based questionnaire to assess the opinion of a panel of Dutch people aged 50 years or older about different aspects of life. To be consistent with other validation studies regarding the WHOQOL-OLD [2, 7, 8, 10], we selected participants of 60 years or older from the panel. In the period December 2009 and January 2010, 1,713 people (≥60 years) completed the ‘Senioren Barometer’; 373 cases were excluded from further analyses because of missing values for quality of life, frailty, and loneliness, yielding a sample size of 1,340.

As we described in previous studies [18, 19], the participants were invited to take part in the study in different ways and through various sources. The first of these was the website www.seniorenbarometer.nl through which people could indicate that they wanted to participate. Organizations for older people in the Netherlands were also asked to issue an announcement of the study on their websites so that persons who were interested in taking part could register. A third major source of participants was computer training courses for older persons given by a large training and educational institute in the Netherlands.

The ‘Senioren Barometer’ 2009/2010 contained the following scales that we used in this study: the WHOQOL-OLD [6], the WHOQOL-BREF [4], the Short Form Health Survey (SF-12) [20], the Tilburg Frailty Indicator (TFI) [21], the Emotional and Social Loneliness Scale (ESLS) [22], and questions regarding socio-demographic characteristics and multimorbidity. In addition, this web-based questionnaire assessed opinions of Dutch people (≥50 years) of the topics of government and municipality, view of life, ageing and care, employment and volunteer work, home and living environment, welfare and health, finance, and leisure and media use.

Medical ethics approval was not necessary as particular treatments or interventions were not offered or withheld from the respondents. The integrity of respondents was not encroached upon as a consequence of participating in this study, which is the main criterion in medical-ethical procedures in the Netherlands [23]. Informed consent, in terms of information-giving and maintaining confidentiality, was respected.

### Measures

#### WHOQOL-OLD

The WHOQOL-OLD is a multidimensional measure of quality of life in older persons and comprises 24 items divided into six facets or subscales of four items each [6]. These facets are sensory abilities, autonomy, past, present, and future activities, social participation, death and dying, and intimacy. The Dutch items were back-translated to English by a native speaker. Then, we provided the same native speaker with the original English items. Although some items were worded differently, the native speaker confirmed the original and back-translated items had the same meaning. Responses were rated on a 5-point Likert scale (1–5), varying in their wording, with higher scores indicating better quality of life. Sum scores were created for each of the six facets and for the WHOQOL-OLD total [6].

#### WHOQOL-BREF

The WHOQOL-BREF comprises 26 items, with 24 items grouped into four domains or subscales (physical health [seven items], psychological [six items], social relations [three items], environment [eight items]), and includes one overall quality of life item and one general health item [4, 24]. Each item was rated on a 5-point scale with a higher score corresponding to a higher quality of life. Domain scores were calculated by multiplying the mean domain score by a factor of 4, resulting in a range from 4–20 for each domain. Only the domain scores were used for analyzing the construct validity of the WHOQOL-OLD.

#### Short form health survey (SF-12)

The SF-12 is a widely used instrument to measure physical and mental aspects of health-related quality of life, which includes 12 items taken directly from the SF-36 [20]. The items have 2–6 response levels and the 12 items are used to derive two summary measures or subscales (i.e. physical and mental component summary) and range from 0–100, in which higher scores indicate better functioning.

#### Tilburg frailty indicator (TFI)

Part B of the TFI, a self-report questionnaire, was used for measuring frailty. Part B comprises fifteen components divided into three domains or subscales - physical (eight items), psychological (four items), and social (three items). The TFI’s total score and the scores for the physical, psychological, and social domains can range from 0–15, 0–8, 0–4, and 0–3, respectively. The maximum scores represent the highest level of frailty. A person is considered as frail when the total TFI score is ≥5 [21]. The TFI has shown good test-retest reliability, good construct validity and good predictive validity for adverse outcomes disability in (instrumental) activities of daily living, indicators of health care utilization, quality of life, and death [21, 25, 26]. The three frailty domain scores were used in the present study. Previous research, using cross-sectional and longitudinal data, showed that the TFI domains are associated with quality of life, measured with the WHOQOL-BREF [18, 27].

#### Emotional and social loneliness scale (ESLS)

The ESLS consists of ten items, measuring emotional loneliness (five items) and social loneliness (five items) [22]. All items are scored on a 5-point Likert scale ranging from 1 (strongly disagree) to 5 (strongly agree). We used the total score for each subscale, which ranges from 5 to 25 with higher scores indicating greater loneliness. Loneliness is a well-known determinant of quality of life [28–30].

#### Background variables: socio-demographic characteristics and multimorbidity

We assessed the following socio-demographic characteristics - sex, age, marital status, highest level of completed education, and the net monthly household income. See Table 1 for a detailed description of the answering categories of these background variables. Multimorbidity was assessed by asking ‘Do you have two or more diseases and/or chronic disorders?’ (yes/no); it has been established that the existence of multimorbidity in individuals is associated with a reduction of quality of life [31–34].

**Table 1:** Participant characteristics (*n* = 1,340)

Characteristic	n (%)
Age, mean ± SD, range	71.0 ± 6.8, 60–95
Sex, % of men	847 (63.2)
Marital status	
Married or cohabiting	950 (70.9)
Single	132 (9.9)
Divorced	73 (5.4)
Widowed	165 (12.3)
Living apart together	20 (1.5)
Education	
None	90 (6.7)
Primary	140 (10.4)
Secondary	587 (43.8)
Polytechnics and higher vocational training	408 (30.4)
University	115 (8.6)
Income^a^	
≤ €999	32 (2.7)
€1000 - €1499	175 (14.9)
€1500 - €1999	227 (19.3)
€2000 - €2499	287 (24.4)
€2500 - €2999	152 (12.9)
€3000 - €3499	133 (11.3)
€3500 - €3999	75 (6.4)
€4000 - €4499	52 (4.4)
> €4499	43 (3.7)
WHOQOL-OLD total and facets, mean ± SD, range	
Total	91.7 ± 10.3, 57–119
Sensory abilities	16.5 ± 2.9, 4–20
Autonomy	15.0 ± 2.1, 7–20
Past, present and future activities	15.2 ± 2.1, 7–20
Social participation	15.5 ± 2.5, 6–20
Death and dying	15.1 ± 3.2, 4–20
Intimacy	14.3 ± 3.1, 4–20
WHOQOL-BREF domains, mean ± SD, range	
Physical health	15.6 ± 1.6, 6.9–20
Psychological	15.1 ± 2.1, 8–20
Social relations	14.2 ± 2.6, 4–20
Environmental	16.0 ± 2.1, 8–20
SF-12 summary measures, mean ± SD, range	
Physical component summary	71.8 ± 23.9, 0–100
Mental component summary	76.2 ± 17.9, 10–100
Frailty domains, mean ± SD, range	
Physical	1.4 ± 1.6, 0–8
Psychological	0.8 ± 1.0, 0–4
Social	0.9 ± 0.9, 0–3
ESLS subscales, mean ± SD, range	
Emotional	11.3 ± 3.8, 5–23
Social	11.5 ± 3.2, 5–24

*SD* Standard Deviation, *SF-12* Short Form Health Survey (SF-12), *TFI* Tilburg Frailty Indicator, *ESLS* Emotional and Social Loneliness Scale

^a^ 164 missing values (12.2 %)

### Analysis strategies

After presenting the descriptive statistics, the results of internal consistency reliability and construct validity analyses were reported. The internal consistency reliability of the six facets of the WHOQOL-OLD and the WHOQOL-OLD total was established using Cronbach’s alpha. Whereas a scale having a reliability coefficient of .6 is sometimes considered sufficient, when the researcher’s goal is to compare groups using that scale [35], a coefficient of .7 is often regarded as being sufficient [36, 37]. What is sufficient however, may depend on context as well as properties of the scale, such as the number of items [36]. Because the WHOQOL-OLD subscales only have four items, we use .6 as a cutoff criterion. The contribution of each item to the reliability of its facet was assessed using the corrected item-total correlation, with coefficients larger than .3 signaling sufficient contribution.

Construct validity was assessed using convergent and divergent validity. Convergent validity was first assessed within the WHOQOL-OLD, using the corrected item-total correlations and Confirmatory Factor Analyses (CFA). Two CFA models were fitted to the data - one uncorrelated 6-factor model with items loading only on their own factor, and a second-order factor model with all six factors also loading on one general factor. The six factors considered in both models were the six WHOQOL-OLD facets sensory abilities, autonomy, past, present, and future activities, social participation, death and dying, and intimacy. Convergent validity within the WHOQOL-OLD is corroborated if both the corrected item-total correlations and factor loadings in the 6-factor model are high (operationalized as larger than .3, following Nunnally and Bernstein [38].

Convergent validity of the WHOQOL-OLD was also determined by examining the correlations between on the one hand the six WHOQOL-OLD facets and on the other hand quality of life measured by four WHOQOL-BREF domains (physical health, psychological, social relations, environmental), the SF-12 physical component summary and mental component summary, the three TFI domains, and the two subscales of the ESLS (emotional loneliness, social loneliness). The TFI and ESLS were included because previous research has shown that quality of life is closely associated with frailty [18, 27, 39] and loneliness [28–30]. Sufficiently high correlations (operationalized as larger than .3) between similar scales support convergent validity. Evidence of divergent validity is obtained if similar scales correlate more strongly than different scales. The following patterns were expected, and hence interpreted as evidence supporting the construct validity:

- WHOQOL-OLD facet sensory abilities has highest correlations with physical measures; WHOQOL-BREF domain physical health, SF-12 physical component summary, TFI physical;
- WHOQOL-OLD facets autonomy, past, present, and future activities, and death and dying have highest correlations with psychological measures; WHOQOL-BREF psychological, SF-12 mental health, TFI psychological;
- WHOQOL-OLD facets social participation and intimacy have highest correlations with social measures; WHOQOL-BREF social relations, TFI social, both subscales of the ESLS.

Finally, we assessed the divergent validity of the WHOQOL-OLD. The six subscales of the WHOQOL-OLD have divergent validity if they measure different constructs. Hence, divergent validity is tested by comparing model fit of the correlated 6-factor model to a (second-order factor) model where all six factors load on one second-order factor. The test of model comparison used was the χ^2^-test [40].

Model fit of both models is assessed using the Root Mean Square Error of Approximation (RMSEA) (.05 and .08 signifying good and reasonable fit, respectively), Comparative Fit Index (CFI), Tucker-Lewis Index (TLI), and Goodness of Fit Index (GFI) (values of .9 and .95 signify satisfactory and good fit, respectively) [40].

Confirmatory factor analyses were carried out using AMOS 22.0, and other statistical calculations were conducted using SPSS 20.0 (SPSS, IBM Corp., Somers, NY, United States of America).

## Results

### Participant characteristics

Table 1 presents the descriptive statistics of the sample. The participants’ mean age was 71.0 years (SD = 6.8); 63.2 % were men and 70.9 % were married or cohabiting.

Applying the cut-off point for frailty identified 27.6 % of the sample as being frail.

### Reliability

Cronbach’s alpha of the WHOQOL-OLD total was .880. Table 2 shows the reliability of the six WHOQOL-OLD facets and the items’ contribution to the reliability of their scale. The six reliabilities all exceeded .655, varying from .656 for autonomy to .909 for intimacy. All corrected item-total correlations exceeded .406, with values of .407-.494 for facets autonomy and past, present and future activities, and higher values for the other four facets, all signifying positive item contributions to the reliabilities of their corresponding facet.

**Table 2:** Reliability (Cronbach’s alpha) of six facets of WHOQOL-OLD, items’ contribution to reliability (corrected item-total correlation), and factor loadings in the 6-factor model

WHOQOL-OLD facet	Cronbach’s alpha	Items no.	Item text	Corrected item total correlation	Factor loading
I: Sensory abilities	.888	1	Impairments to senses affect daily life	.780	.750
		2	Loss of sensory abilities affect participation in activities	.822	.782
		10	Problems with sensory functioning affect ability to interact	.725	.658
		20	Rate sensory functioning	.703	.548
II: Autonomy	.656	3	Freedom to make own decisions	.482	.274
		4	Feel in control of your future	.483	.522
		5	People around you are respectful of your freedom	.440	.351
		11	Able to do things you’d like to	.409	.576
III: Past, present and future activities	.674	12	Satisfied with opportunities to continue archiving	.407	.459
		13	Received the recognition you deserve in life	.490	.419
		15	Satisfied with what you’ve achieved in life	.494	.401
		19	Happy with things to look forward to	.438	.382
IV: Social participation	.804	14	Have enough to do each day	.522	.495
		16	Satisfied with the way you use your time	.689	.581
		17	Satisfied with level of activity	.704	.611
		18	Satisfied with opportunity to participate in community	.577	.580
V: Death and dying	.823	6	Concerned about the way you will die	.707	.722
		7	Afraid of not being able to control death	.691	.858
		8	Scared of dying	.632	.681
		9	Fear pain before death	.576	.623
VI: Intimacy	.909	21	Feel a sense of companionship in life	.712	.617
		22	Experience love in your life	.819	.717
		23	Opportunities to love	.815	.798
		24	Opportunities to be loved	.829	.833

### Construct validity

The convergent validity within the WHOQOL-OLD is corroborated by sufficient corrected item-total correlations (all larger than .406) and generally sufficient item loadings on their own factor (with the exception of one item of the sensory abilities scale, which had a loading of .274, all loadings were larger than .350) (see Table 2, last two columns). Correlations of WHOQOL-OLD with other scales generally also support its convergent validity; the five WHOQOL-OLD scales correlate more strongly than ± .3 with similar scales, with the exception of one correlation of the scale death and dying (with the mental health subscale of the SF-12 [.261]) and one correlation of the scale social participation (with the social domain of the TFI [−.284]). Because deviations of expectations both within and between scales were few and minor, we conclude that the WHOQOL-OLD has good convergent validity.

The divergent validity of four WHOQOL-OLD subscales was supported, but results were mixed for two of the subscales. More specifically, as expected the subscale sensory abilities correlated more strongly with similar scales (WHOQOL-BREF physical health, SF-12 physical component summary, TFI physical) than with dissimilar scales. That is, the correlations printed in bold in Table 3 exceed the other correlations in the column of the subscale sensory abilities. The two ‘psychological’ WHOQOL-OLD subscales past, present, and future activities and death and dying also show strongest correlations with similar scales (WHOQOL-BREF psychological, SF-12 mental component summary, TFI psychological). The ‘social’ WHOQOL-OLD subscale intimacy also showed good divergent validity, as it correlated strongest to its most similar scales (WHOQOL-BREF social relations, TFI social, the ESLS scales). Divergent validity of the autonomy and social participation subscales was not fully supported. The ‘psychological’ facet autonomy correlated more strongly with the TFI physical domain and the environmental WHOQOL-BREF subscale than with the psychological subscales of the TFI and WHOQOL-BREF. The ‘social’ participation subscale correlated more strongly with the other WHOQOL-BREF and TFI subscales than with the social WHOQOL-BREF and social TFI scales. Moreover, the ‘psychological’ past, present and future facet correlated as strongly with the two loneliness (ESLS) scales as the social participation and intimacy facets.

**Table 3:** Correlations WHOQOL-OLD (facets, total) with WHOQOL-BREF, SF-12, TFI, and ESLS.^a, b^ and (Cronbach’s alpha) reliabilities of subscales

	Cronbach’s alpha	Sensory abilities	Autonomy	Past, present and future activities	Social participation
WHOQOL-BREF					
Physical health	.841	**.409**	.507	.518	.542
Psychological	.758	.336	**.536**	**.676**	.585
Social relations	.611	.178	.344	.526	**.459**
Environmental	.792	.361	.576	.595	.517
SF-12					
Physical component summary	.859	**.387**	.427	.413	.430
Mental component summary	.779	.385	**.484**	**.536**	.559
TFI					
Physical	.673	**−.509**	−.367	−.344	−.377
Psychological	.567	−.262	**−.354**	**−.434**	−.365
Social	.506	−.119	−.105	−.307	**−.284**
ESLS					
Emotional loneliness	.704	−.131	−.168	−.338	**−.319**
Social loneliness	.744	−.170	−.282	−.432	**−.416**
	Death and dying	Intimacy	WHOQOL-OLD total		
WHOQOL-BREF					
Physical health	.261	.283	.621		
Psychological	**.308**	.434	.709		
Social relations	.204	**.536**	.563		
Environmental	.246	.352	.647		
SF-12					
Physical component summary	.199	.214	.510		
Mental component summary	**.261**	.350	.637		
TFI					
Physical	−.183	−.206	−.498		
Psychological	**−.332**	−.287	−.511		
Social	−.137	**−.563**	−.398		
ESLS					
Emotional loneliness	−.105	**−.670**	−.451		
Social loneliness	−.126	**−.442**	−.465		

*SF-12* Short Form Health Survey (SF-12), *TFI* Tilburg Frailty Indicator, *ESLS* Emotional and Social Loneliness Scale

^a^ All *p* < .001

^b^ Correlations expected to be high are printed in bold

Support of divergent validity of the WHOQOL-OLD is provided by the comparison of the fit of the 6-factor model and the second-order factor analysis model. The fit of the 6-factor model was significantly better (χ^2^(10) = 90.45, *p* < .001) than of the second-order factor model. Fit indices of the 6-factor model were RMSEA = .063 (90 % CI .060-.066), GFI = .912, TLI = .903, CFI = .917, suggesting reasonable to good fit. The fit indices for the second-order factor model were only slightly worse; RMSEA = .064 (90 % CI .061-.067), GFI = .905, TLI = .901, CFI = .912). The standardized regression weights of individual items on the factors (comparable to factor loadings) were at least .51 in both models.

Table 4 shows the correlations between the WHOQOL-OLD facets (upper-right), and their corresponding factors (lower-left). Remarkable are the extremely high correlations between factors autonomy and past, present and future activities (.938), autonomy and social participation (.704), and past, present and future activities and social participation (.877). These extremely high correlations suggest that these three subscales are closely related, suggesting low divergent validity of these three subscales.

**Table 4:** Correlations between facets WHOQOL-OLD, and their corresponding factors^a,b^

Facet	Sensory abilities	Autonomy	Past, present and future activities	Social participation	Death and dying	Intimacy
Sensory abilities		.334	.299	.288	.138	.174
Autonomy	.445		.595	.523	.227	.230
Past, present and future activities	.397	.938		.642	.282	.438
Social participation	.303	.704	.877		.201	.371
Death and dying	.147	.294	.372	.235		.176
Intimacy	.187	.297	.538	.408	.206	

^a^ All *p* < .001

^b^ Correlation between scale scores in upper right, correlation between factors in the lower left

## Discussion

The present study examined the psychometric properties of the Dutch version of the WHOQOL-OLD, using a large sample of 1,340 people aged 60 years or older who completed the web-based questionnaire ‘Senioren Barometer’. Simultaneously with the WHOQOL-OLD, participants completed the WHOQOL-BREF, Short Form Health Survey (SF-12), Tilburg Frailty Indicator (TFI), Emotional and Social Loneliness Scale (ESLS), and questions on socio-demographic characteristics and multimorbidity.

The reliabilities of the six WHOQOL-OLD facets and WHOQOL-OLD total were sufficient to good. Although subscales autonomy and past, present and future activities had reliabilities in the range .65-.70, the contribution of all their items to the reliability was sufficient as shown by their corrected item-total correlations. Bearing in mind the scales are short having only four items, a reliability of about 0.6 or more is reasonable. Because lower reliabilities attenuate associations [41], this implies that somewhat higher sample sizes are needed to establish associations with the two facets autonomy and past, present, and future activities, than with the four other facets.

We concluded that the convergent validity of the WHOQOL-OLD was good, both concerning associations within the WHOQOL-OLD and associations with other scales, because deviations of expectations were few and minor. Our findings on the divergent validity of the WHOQOL-OLD were somewhat mixed. Findings corroborating the divergent validity were that the 6-factor model fitted better than the second-order factor model, and four facets (sensory abilities, past, present and future activities, death and dying, intimacy) correlated more strongly with similar than dissimilar scales. However, several findings did not fully support divergent validity, and these we discuss one by one.

First, the ‘psychological’ facet autonomy correlated more strongly with the TFI physical domain and the environmental WHOQOL-BREF subscale than with the psychological subscales of the TFI and WHOQOL-BREF. Our interpretation of this finding is that the autonomy facet is not only psychological, but also taps into other domains such as the physical and environmental domain. Someone’s autonomy will depend to a very large extent on physical ability and the support opportunities that the environment has to offer [42, 43].

Second, the ‘social’ facet, social participation correlated more strongly with the other WHOQOL-BREF and TFI subscales than with the social WHOQOL-BREF and social TFI scales. We examined if after correction for the lower reliabilities of the social subscales, by application the correction for attenuation formula [44], correlations were as expected (highest correlations of social participation with the social subscales). However, after applying the correction, social participation correlated most strongly with the psychological subscales of the WHOQOL-BREF and TFI. Hence we conclude that the social participation facet taps more into the psychological than the social domain. If we further consider the four items of the facet social participation, our finding is less surprising. All items refer to satisfaction regarding opportunities for activities, while the social WHOQOL-BREF and the social TFI scale are more focused on maintaining social relations.

Finally, although the 6-factor model fitted better than the second-order factor model, the factors corresponding to autonomy, past, present and future activities, and social participation were correlated extremely strongly (correlations of .704 to .938). Our findings are in line with the German version of the WHOQOL-OLD, who found correlations of .808 to .907 between these factors [2]. These findings suggest that these three facets measure more or less the same, mostly psychological dimension. Future research could examine their interrelations, and whether using the three individual facets in future research is justified or not.

Frailty domains (physical, psychological, social) were associated with all WHOQOL-OLD facets, with coefficients larger than .3, confirming that all three frailty domains are relevant for predicting all quality of file domains [18, 27].

Loneliness is known to negatively affect quality of life in old age [28–30]. In our study loneliness was associated with all facets of the WHOQOL-OLD. Social loneliness refers to the absence of an acceptable social network, whereas emotional loneliness refers to the absence of an attachment figure in one's life and someone to turn to [45]. The concept of emotional loneliness is particularly important for older adults [46]. Older people living alone must be evaluated as a high-risk group; policy-makers and healthcare and welfare professionals should be aware of the factors that can lead to loneliness such as being widowed, advancing age, low level of education and income, low self-esteem, and poor health [47, 48]. Interventions to reduce loneliness should target these groups particularly, and promote activities that have the potential to enhance quality of life.

The present study has some limitations. Due to the cross-sectional design of our study, it was not possible to assess the test-retest reliability of the Dutch version of the WHOQOL-OLD. Other limitations include the possibility of selection bias of the participants due to the exclusion of people without Internet access. In addition, we are lacking information whether the study participants are community-dwelling or residents of assisted living facilities. These two groups show rather large differences in variables related to frailty and quality of life, with residents of assisted living facilities scoring higher on frailty and lower on quality of life [49]. However, we have no reasons to suppose that associations between variables related to quality of life are different for these two groups. Hence we also have no reasons to suppose that group composition will have affected our results.

The results of our study reveal that the psychometric properties of the Dutch version of the WHOQOL-OLD are similar than those reported from the international WHOQOL-OLD field study [6] and of other county versions [2, 7–11]. A good construct validity as shown by high factor loadings in the 6-factor model was also obtained for the German and Chinese versions of the WHOQOL-OLD [2, 10]. These results emphasize the multidimensional nature of quality of life.

## Conclusion

We offer Dutch healthcare and social workers an instrument with good psychometric properties for measuring quality of life in older people. We recommend that professionals use the WHOQOL-OLD instead of other existing quality of life measures because it encompasses subscales particularly relevant for older people (autonomy, death and dying, intimacy). This instrument can be used in addition to the WHOQOL-BREF, and also separately, especially when aiming to assess more psychological aspects of quality of life in older people.
